# Linking B cell metabolism to immune regulation: the neurotransmitter GABA

**DOI:** 10.1038/s41392-022-01120-w

**Published:** 2022-08-01

**Authors:** Yang Shi

**Affiliations:** Institute for Experimental Molecular Imaging, Uniklinik RWTH Aachen and Helmholtz Institute for Biomedical Engineering, Faculty of Medicine, RWTH Aachen University, Aachen, 52074 Germany

**Keywords:** Lymphocytes, Tumour immunology

In a recent study published in Nature,^1^ Zhang et al. reported that activated B cells produced and released the principal inhibitory neurotransmitter γ-aminobutyric acid (GABA). The GABA secretion was demonstrated to inhibit cancer immunity by suppressing cytotoxic T cells and macrophages. The study revealed a new immune regulation mechanism of B cells, which suggests a new direction to understand cancer immunity and develop immunotherapeutics.

Our B cells have been under the spotlight in the past few years. For example, B cells differentiate into plasma cells which produce antibodies against SARS-CoV-2 during viral infection and vaccination. Antibody production is only one of the many immunological functions of B cells. In immuno-oncology, B cells’ importance is getting increasingly recognized.^2^ Although B cell immunity in cancer is not fully understood yet, it is at least already known that B cells’ roles are two-sided: they can be anti-tumor and pro-tumor. B cells can contribute to cancer immunotherapy via humoral and cellular immune responses. B cells differentiate into plasma cells to produce cancer specific antibodies. Moreover, B cells seem to play important roles in the formation and functions of tertiary lymphoid structures in solids tumors, which have shown significant correlations with favorable prognosis and better survival of patients after immunotherapy. However, B cells can be immunosuppressive and this is crucial in determining B cells’ overall contribution to cancer immunity.

**Fig. 1:**
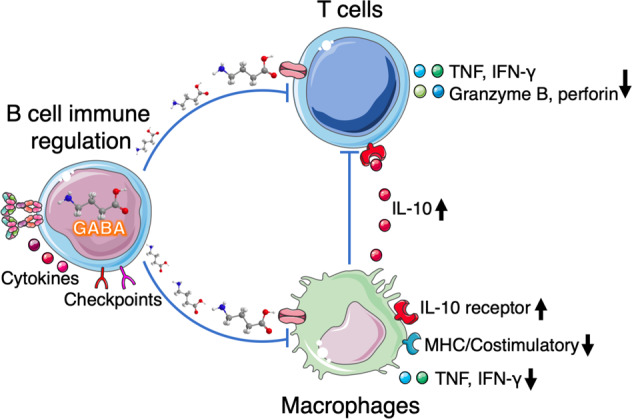
B cell-mediated immune regulation via the production and release of GABA. Previously, B cells have been reported to show immunoregulatory phenotypes including the secretion of anti-inflammatory cytokines (e.g., IL 10 and transforming growth factor beta (TGF-β)) and surface expression of checkpoint proteins (e.g., PD-1/L1). The current study^1^ demonstrated that activated B cells can also produce the neurotransmitter GABA to impair the anti-tumor capability of T cells and macrophages. GABA directly inhibited T cell activation by downregulating inflammatory cytokines (e.g., TNF and IFN-γ) and cytotoxic proteins (e.g., granzyme B and perforin). Moreover, GABA induced anti-inflammatory tumor-associated macrophages which released IL-10 and this suppressed T cell activation. The figure elements are available from smart.servier.com

The study began with non-targeted profiling of water-soluble small molecule metabolites in mice after adjuvanted protein vaccination in the footpad. Strong difference in metabolic pathways was observed between the non-draining and draining lymph nodes, especially those related with alanine, aspartate and glutamate. This study was repeated in three genetically modified mouse models that lack B cells, T cells or both B and T cells to track which population of lymphocytes was the major contributor to the metabolic shift. It became clear that B cells were mainly responsible for the alteration of the immune metabolism and, for the first time, B cells were found to produce high levels of GABA, the well-known neurotransmitter. Furthermore, B cells in different tissues and differentiation status, both in human and mice, were characterized with high levels of GABA. A related finding was that decarboxylase 67, a key enzyme for GABA synthesis, was significantly more expressed in B cells than in T cells. In vitro assays showed that B cells upon activation via different modes of stimulation (agonistic ligation of pattern recognition receptors and crosslinking of B cell receptors) produced GABA. Subsequently, immunological functions of B cell-released GABA in cancer models was investigated. Compared to wide-type mice, B cell knockout mice had slower tumor growth which could be elevated by implanting materials releasing GABA. Furthermore, tumor infiltrating CD8^+^ T cells in GABA-treated mice showed downregulation of tumor necrosis factor (TNF) target gene transcripts. By pharmacologically blocking GABA_A_ receptors, several effector functions of cytotoxic T cells were restored. In an in vitro cytotoxic T cell activation assay, GABA or a GABA_A_ receptor agonist reduced T cell activation or lowered the release of inflammatory cytokines. These results clearly demonstrated that GABA produced by B cells or the GABA_A_ signalling were critical in suppressing CD8^+^ T cells. Afterwards, another important tumor-infiltrating immune cells, macrophages, were examined in the context of GABA secretion by B cells. Firstly, the authors showed that the contribution of macrophages to tumor growth control was different in the presence or absence of B cells. Then, exogeneous GABA was introduced in B cell knockout mice and it significantly downregulated the gene transcription profiles related to inflammatory cytokines such as TNF and interferon-γ (IFN-γ) in tumor-associated macrophages. GABA treatment activated macrophage energy metabolism pathways including OXPHOS and PPAR signalling and downregulated nitric oxide and ROS production pathways. By co-treating macrophages with GABA and interleukin (IL)-10, transcripts of cytokines, cytokine receptors and major histocompatibility complex presentation pathway molecules were downregulated and that of IL-10 was upregulated (Fig. 1). These pointed to the induction of anti-inflammatory immune phenotypes, which could be partially reverted by blocking the GABA_A_ signalling pathway. Finally, the authors constructed a genetically modified mouse model that allowed for specific downregulation of GABA production by B cells. MC38 colon tumours inoculated in this mouse model showed much slower growth and infiltration of T cells with cytotoxic and inflammatory phenotypes.

Overall, the current study revealed a new mechanism of B cell-mediated immunosuppression via GABA production. GABA is the first discovered small molecule immunosuppressive factor produced by B cells. Previously known immunosuppressive soluble factors produced by B cells are proteins, i.e., TGF-β, IL-10 and IL-35.^3^ All these discoveries together support the hypothesis that B cells often develop immuno-regulatory phenotypes upon activation. Initial research on regulatory B cell can be dated back to two decades ago. Now, we know that different stimuli lead to different immunosuppressive phenotypes of B cells. However, it is still difficult to correlate specific regulatory phenotypes of B cells with the stimulation. This adds significantly to the complexity of B cell immunity in immuno-oncology since cancer treatment is often based on combinations of different (immuno)therapeutics. Moreover, it is obviously an important question how to deal with the immunosuppressive functions of B cells in cancer treatment. This is probably among the biggest hurdles in attempts to harness B cell immunity in immunotherapy. One strategy could be optimizing the combination and strength of stimuli to avoid inducing strong immunosuppression by B cells. Furthermore, the immunosuppressive functions of B cells can be pharmacologically or genetically inhibited. Both approaches have been demonstrated feasible to control GABA production by B cells in the current study. Our group has also proven that B cells can be treated with specific inhibitors to suppress the biosynthesis of macromolecular immunosuppressive factors during activation (unpublished data). These efforts suggest that there are great opportunities in promoting anti-cancer B cell immunity by employing inhibitors delivered via engineered materials.^4^ By further deepening our understanding of regulatory B cells and designing therapeutic approaches to address this issue, the enormous potential of B cells will be optimally utilized for cancer treatment.
